# Macrophage activation syndrome in a patient with pulmonary inflammatory myofibroblastic tumour

**DOI:** 10.1186/1710-1492-8-6

**Published:** 2012-05-20

**Authors:** Christoph Kuppe, Saskia Westphal, Eva Bücher, Marcus J Moeller, Bernhard Heintz, Marion E Schneider, Jürgen Floege

**Affiliations:** 1 Department of Nephrology and Clinical Immunology, University Hospital of the Aachen University of Technology (RWTH), Aachen, Germany; 2 Department of Pathology, University Hospital of the Aachen University of Technology (RWTH), Aachen, Germany; 3 Department of Experimental Anesthesiology, University Hospital, Ulm, Germany

**Keywords:** Macrophage activation syndrome, Haemophagocytosis, Pulmonary plasma cell granuloma

## Abstract

We describe for the first time a case of macrophage activation syndrome (MAS) in a patient with a history of inflammatory myofibroblastic tumour (inflammatory pseudotumour, IPT) of the lung and thoracic spine. The patient was admitted to the intensive care unit with a history of prolonged remitting fever, hepatosplenomegaly, bilaterally enlarged thoracic lymph nodes and an acute severe inflammatory response syndrome (SIRS). Up-regulated cytokine production (e.g. IL-1ß and IL-6), increased levels of ferritin and circulating soluble interleukin-2 receptor (sIL-2R, sCD25) led to the differential diagnosis of MAS. Bone marrow aspiration, the main tool for a definite diagnosis, revealed macrophages phagocytosing haematopoietic cells. Immunosuppressive therapy with corticosteroids and cyclosporine was an effective treatment in this patient.

## Introduction

Macrophage activation syndrome (MAS) is a rare but potentially fatal disorder, characterized by combinations of pancytopoenia, liver failure, coagulopathy and organ dysfunction. It is thought to be caused by the activation and uncontrolled proliferation of CD8^+^ lymphocytes and well-differentiated macrophages, leading to haemophagocytosis and a so-called cytokine storm [1-3]. The term MAS describes a condition occurring in a broad spectrum of diseases, which belong to the histiocytic disorders, e.g. haemophagocytic lymphohistiocytosis (HLH). Familial or primary haemophagocytic lymphohistiocytosis has a known and well-characterized genetic basis, namely a mutation in the perforin gene [4]. It results in the inability of cytotoxic T lymphocytes (CTLs) or natural killer (NK) cells to lyse target cells. Secondary HLH can be found in patients with infections, malignancies and inflammatory diseases such as juvenile idiopathic arthritis (JIA) or it may be an adverse effect of certain drugs [5].

Inflammatory myofibroblastic tumours are rare tumours with different invasive tendencies and growth capacity [6]. In fact, this tumour entity represents the non-neoplastic unregulated growth of inflammatory cells and fibroblasts or myofibroblasts, irrespective of their organs of origin [7].

Pulmonary inflammatory myofibroblastic tumours usually consist of a mixed inflammatory infiltrate with a predominance of plasma cells [8]. Two theories exist concerning the origin of IPTs. According to one theory, the tumour is in fact a regular inflammatory process, which follows interstitial pneumonia. Subsequently, it transforms into an organized pneumonia and, eventually, into an inflammatory myofibroblastic tumour. In support of this hypothesis, production of IL-6 mRNA and protein by tumour cells in IPT has been reported in some cases [8,9]. Yet, according to another hypothesis, some plasma cell granulomas represent slow-growing mesenchymal tumours with secondary inflammatory changes.

In this report, we describe for the first time the occurrence of MAS in a patient with a history of an inflammatory myofibroblastic tumour of the lung and thoracic spine.

## Case report

**Figure 1:**
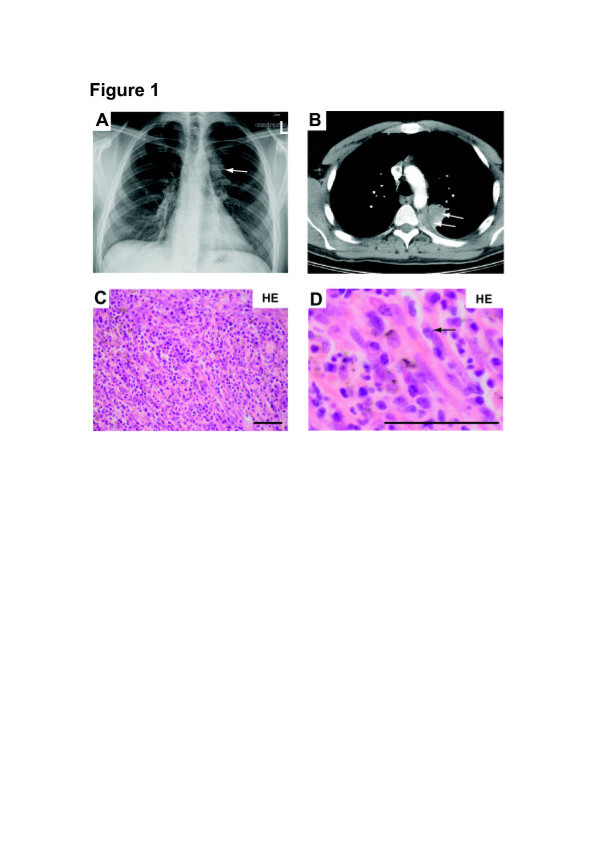
**Aspects of the pseudoinflammatory tumour of the lung in the patient.** ( **A**) A chest radiography revealed a mass in projection of the upper left lung lobe. ( **B**) A computed tomography of the lung revealed a mass in the left lobe of the lung in contact with the thoracic spine. ( **C**- **D**) Histological analysis revealed the diagnosis of a pseudoinflammatory tumour, with the characteristic spindle-like cell structure of fibroblasts.

The patient, a 27 year-old male, presented initially to his general practitioner because of increasing back pain. As an electrical mechanic he traveled extensively from Germany to China, Russia and South Korea and was mainly involved in the installation of electronic devices in newly built tunnels. Due to back pain and an evolving neurological deficit of the lower extremities, the patient was admitted to an external orthopaedic department. A magnetic resonance imaging (MRI) of the spine revealed an atypical tumour in the left upper lung lobe encasing the vertebrae Th3-Th4. A computerized tomography (CT) of the thorax revealed a mass of 3.9 cm x 2.9 cm in the left upper lung lobe (Figure1A, B, arrows). Histological examination of a biopsy of the mass showed an IPT with large amounts of histiocytes, lymphocytes and plasma cells (Figure1C, D). After neurosurgical therapy, the neurological deficit in the lower extremities improved and the patient was discharged. In a second operation, the lung tumour was removed. Histological examination of this material also revealed an IPT. Given the extensive travel history of the patient, various infectious agents were excluded (mycobacteria, legionella, Borrelia burgdorferi, pneumocystis, Epstein-Barr virus (EBV), herpes simplex virus, human immunodeficiency virus-1/2, parvovirus, respiratory viruses, cytomegalovirus (CMV) and other bacteria, stool pathogens and urinary histoplasma antigen). During the following two years, the patient was intermittently treated with corticosteroids, and low-dose radiation therapy was performed in order to control tumour growth locally, as recommended previously [10].

**Figure 2:**
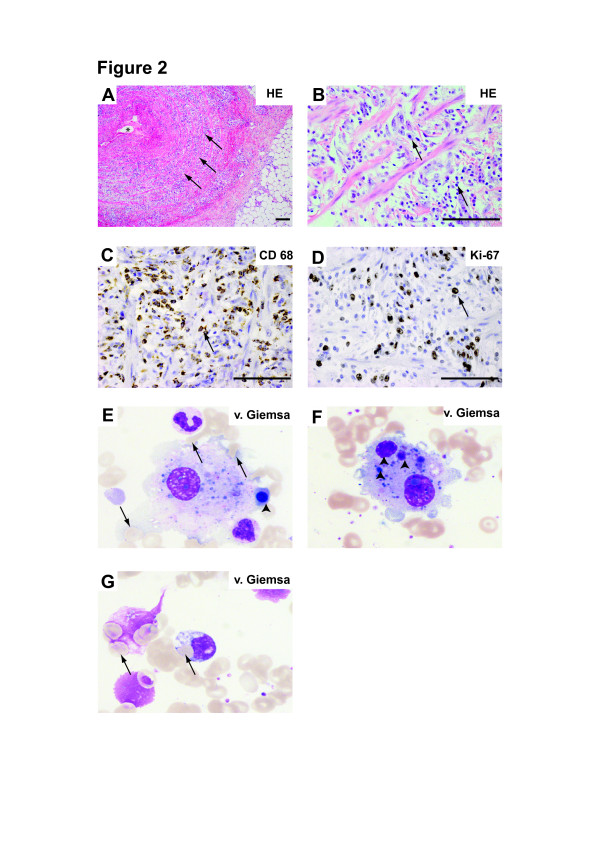
**Routine and immunohistochemical stainings of biopsies from cubital veins and bone marrow.** ( **A**- **B**) Biopsies of cubital veins showed complete loss of normal vein architecture with massive infiltration of lymphocytes (arrows in **A**), histiocytes (arrows in **B**), macrophages and fibroblasts. ( **C**- **D**) Further immunohistochemical analysis revealed massively increased numbers of proliferating Ki-67^+^/CD68^+^ macrophages (arrows in **C** and **D**). ( **E**- **G**) Bone marrow biopsies of the patient revealed haemophagocytosis, the defining criterion of MAS. Characteristically, the histiocytes showed degenerated and nucleated proerythoblasts within their cytoplasm (arrows **E**- **G**).

After two-and-a-half years, the internal fixator was removed without complications. A few days later, the patient presented to a general practitioner because of dizziness and mild fever. The operation wound appeared normal and laboratory tests revealed no evidence of infection. During the following two weeks, the symptoms increased and the patient was admitted to the hospital. Postoperative spondylodiscitis was ruled out by CT and MRI of the spine. The CT scan revealed enlarged mediastinal lymph nodes and hepatomegaly. Subsequently, he developed SIRS and was admitted to the intensive care unit. Blood cultures, initial bone marrow aspiration as well as a biopsy and transesophageal echocardiography all came back negative or normal. Histologic examination of material obtained during mediastinoscopy as well as a bronchial lavage showed no malignant cells; the CD4/CD8 ratio was not elevated. No granulomas were observed and sarcoidosis thus appeared unlikely. Subsequently, both elbows started to appear swollen and hot. A biopsy ruled out fasciitis but immunohistopathological evaluation showed histiocytic inflammation with CD68-positive foamy histiocytes and absence of CD1a immunoreactivity (excluding a Langerhans cell histiocytosis) (Figure2 A-C). A liver biopsy was performed; the tissue showed no signs of sarcoidosis and only a mild non-specific inflammatory response. Additionally, there was no evidence for a malignant B- or T-cell lymphoma in subclonal tests using semi-nested PCRs of the biopsy material from the lungs. A positron emission tomography (PET) CT scan showed no hypermetabolic region. Finally, histological evaluation of a second bone marrow biopsy showed haemophagocytic histiocytes (Figure2, E-G).

**Figure 3:**
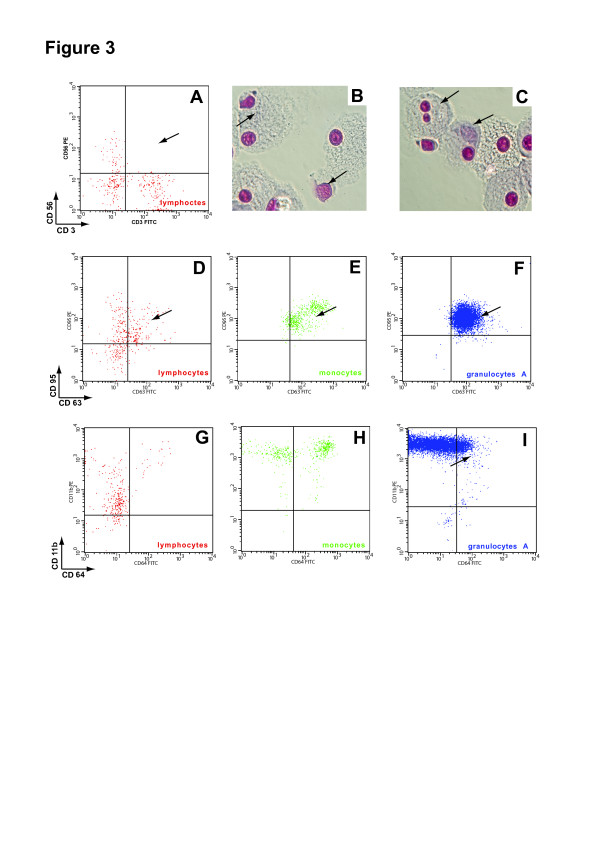
**Flow cytometric analysis of bone-marrow cells and morphology of antigen-presenting cells (APC) from long-term-cultures.** ( **A**) NKT (CD3^+^/CD56^+^ cells) were no longer present in the bone marrow of the patient. (**B**- **C**) Images show Giemsa-stained cytospin preparations of 28-day-cultured haemophagocytes with characteristic autophagy vacuoles and inclusion bodies containing undigested phagocytozed material (arrows). ( **D**- **I**) Further FACS analysis revealed a high expression of CD63 on all BMCs and also an increased expression of CD11b on granulocytes.

Flow cytometric analysis revealed the absence of NKT cells and an increased expression of CD163 on all bone marrow cells. CD163, a receptor for haemoglobin-haptoglobin complexes, is involved in clearance and endocytosis of haemoglobin/haptoglobin complexes by macrophages and may thereby protect tissues from free haemoglobin-mediated oxidative damage. The plasma levels of soluble CD163 in HLH are considerably higher than those found in infections or autoimmune diseases [11]. In the peripheral blood, we also found a slight increase of the relative amount of B-lymphocytes as well as an increased expression of CD63 (degranulation marker) and CD11b (migration marker on neutrophils) on bone marrow cells (Figure3, A-I).

One week after admission, an immunosuppressive regimen was started including 1 mg/kg body weight methylprednisolone and 5 mg/kg body weight ciclosporin. The fever decreased over several days after initiation of therapy and laboratory values normalized (Table1). After two weeks of therapy, the cytokine levels in the peripheral blood were already dramatically reduced (Table2). The patient was maintained on 5 mg prednisolone and cyclosporine 2-6 mg/kg/d and to date he has had no further relapses for over a year.

**Table 1:** Laboratory Values

**Variable**	**fever episode**	**after 2 weeks**	**after 5 months**	**normal range**
Haematocrit (%)	0,25	0,34	0,43	0.4–0.54
White-cell count (10^6/l)	15,10	13,10	11,9	4.3–10.0
Platelet count (10^6/l)	587	519	235	150–350
Ferritin (μg/l)	1043	230	204	30–400
Triglycerides (mg/dl)	68	127	208	< 200
IL2R (kU/l)	> 7200	2000	690	223–710
IL6 (ng/l)	1500	250	n.d.	< 7
CRP (mg/l)	>230 +	53	10	< 5
PCHE (U/l)	918	3218	9495	5320–12920
Prothombin time (INR)				0.8–1.1
Fibrinogen (g/l)	4,8	n.d.	n.d.	2.0–4.5

**Table 2:** Diagnostic criteria for HLH*

**(1) Fever**	Manifestations in the patient described quotidian fever over several weeks
**(2) Splenomegaly**	hepatosplenomegaly
(3) Cytopenia involving two or more cell lines	haemoglobin < 9.0 d/dL, no other
(4) Hypertriglyceridaemia or hypofibrinogenaemia	no
**(5) Haemophagocytosis**	yes
**(6) Hepatitis**	yes
**(7) Low or absent natural killer cell activity**	yes
**(8) Serum ferritin level > 500 μg**	1043 μg/L
**(9) Soluble CD25 (sIL-2 receptor) > 2500 U/ml**	> 7200 U/ml

*according to HLH-2004 criteria (Henter JI et al.).

## Discussion

In acquired cases of macrophage activation syndrome (MAS), the clinical course is usually rapidly progressive with multi-system organ failure often occurring within weeks of the initial diagnosis of the syndrome. This syndrome is caused by dysregulated macrophage-lymphocyte interaction, which leads to uncontrolled proliferation of macrophages and CD8^+^ T-cells with up-regulated release of monokines, mainly of the interleukin-1 family (interleukin-1α and 1β and interleukin-18), whereas levels of T-cell–derived cytokines, such as interferon-γ, are much less increased [12]. Recently, cell-type specific characteristics of ‘immature antigen presenting cells (APC)’ from patients with MAS have been described. It is known that tumours (especially fibroblasts) can express M-CSF, which could influence the development of immature phagocytic APCs [13]. In our patient, fulminant MAS was present for approximately 3 weeks until the initiation of therapy. The standard definition of HLH requires the presence of at least five of nine clinical criteria. Our patient fulfilled seven of these criteria (Table2). Additionally, we could find an extramedullary manifestation of MAS, namely a proliferative (Ki-67-positive) histiocytic cell infiltrate in both elbows of the patient. Typically in the acute phase of the cytokine storm, lymphohistiocytic infiltrates can be found in the spleen, lymph nodes and bone marrow [5]. A CD1a staining of the cubital veins remained negative, thus making the diagnosis of Langerhans-cell histiocytosis unlikely. In the absence of a clinically apparent malignancy, in addition to MAS, the differential diagnosis for fever with splenomegaly, liver failure and bilaterally enlarged lymph nodes includes premalignant, inflammatory, infectious, genetic and toxic causes. All of these could be ruled out on the basis of the history and laboratory studies. Acute EBV and CMV infections are associated with fever, pharyngitis, lymphadenopathy and fatigue and would likely have been self-limited. The differential diagnosis of sarcoidosis was mainly ruled out because no granulomas could be found in any of the biopsies. Furthermore, the bone marrow did not show any premalignant, infiltrative or infectious processes.

Several treatment options have been reported in the literature for MAS. A treatment protocol from the Histiocyte Society recommends a therapeutic regimen of etoposide, dexamethasone and cyclosporine [14]. In other cases, high-dose steroids, cyclosporine, antihuman thymocyte globulin (ATG), intravenous immune globulin (IVIG), plasma exchange and allogeneic bone marrow transplantation have been described. Recently, treatment with an IL-1 beta receptor antagonist (anakinra) was successful in several cases of severe paediatric rheumatic disease-associated MAS [15,16]. It can be speculated that other immunomodulatory therapies (alemtuzumab, infliximab, daclizumab, selective IL-1 and IL-6 antagonists) might influence the course of the disease beneficially, but clinical trials remain almost impossible due to the rare occurrence of the disease.

In summary, we describe the first patient with macrophage activation syndrome (MAS) and a history of an inflammatory myofibroblastic tumour, which could be controlled by immunosuppressive therapy including steroids and cyclosporine.

## Consent

Written informed consent was obtained from the patient for publication of this case report.

## Competing interests

The authors declare that they have no competing interests.

## Authors’ contributions

BH and JF diagnosed the case and planned the treatment and medical follow up. CK gathered the patient's history and drafted the manuscript and the subsequent revisions. MJ and JF participated in manuscript revision. SW carried out immunohistochemistry. MS carried out FACS and in-vitro studies. All authors read and approved the final manuscript
